# Supplementary material for: Identification of photoperiod-induced specific miRNAs in the adrenal glands of Sunite sheep (Ovis aries)
Source: Front Vet Sci. 2022 Jul 22;9:888207. doi: 10.3389/fvets.2022.888207 (PMC9354845; doi:10.3389/fvets.2022.888207)
Supplement: Supplementary file 1 [file Data_Sheet_1.ZIP › Supplementary file/miRDeep2 file.zip!/miRDeep2 file/Novel_Structure/LP42A2/Novel_667.pdf]

Provisional ID	:	NC_019481.2_35386
Score total	:	3664.6
Score for star read(s)	:	3.9
Score for read counts	:	3657.1
Score for mfe	:	2
Score for randfold	:	1.6
Score for cons. seed	:	
Total read count	:	7185
Mature read count	:	7175
Loop read count	:	0
Star read count	:	10

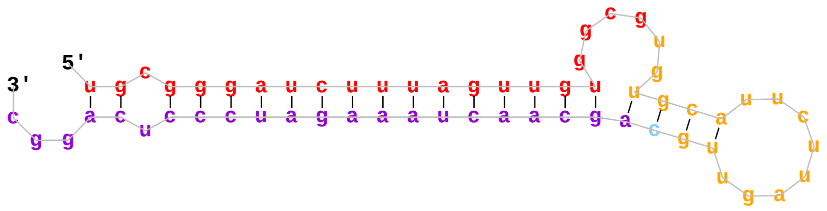

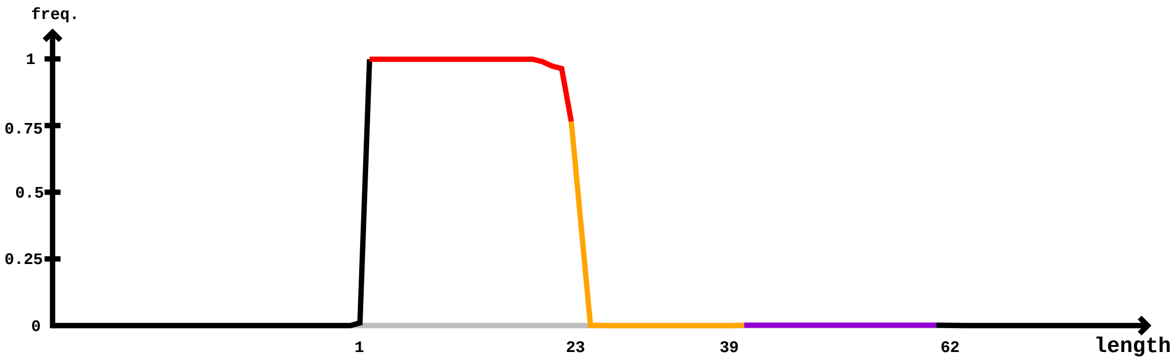

Mature Star

```
5'- auaacccauaaaaagcagccgacguugcggcaugcgggaucuuuaguuguggcgugugcauucuuaguugcagcaacuaaagaucccucaggcugcaaugaagaucagagauc -3'   obs
    auaacccauaaaaagcagccgacguugcggcaugcgggaucuuuaguuguggcgugugcauucuuaguugcagcaacuaaagaucccucaggcugcaaugaagaucagagauc        exp
    ......................((((((((.((.((((((((((((((((......((((........))))))))))))))))))).)).)))))))))..((((...))))   reads   mm   sample
    ...............................augcgggaucuuuaguugC...............................................................   1       1    seq
    ...............................augcgggaucuuuaguugug..............................................................   4       0    seq
    ...............................augcgggaucuuuaguugugU.............................................................   2       1    seq
    ...............................augcgggaucuuuaguugugg.............................................................   14      0    seq
    ...............................auUcgggaucuuuaguugugg.............................................................   1       1    seq
    ...............................augcgggaucuuuaguugCgg.............................................................   1       1    seq
    ...............................augcgggaucuuuagCuguggc............................................................   1       1    seq
    ...............................augcgggaucuuuaguuguggU............................................................   24      1    seq
    ...............................augcgggaucuuuaguuguggcA...........................................................   20      1    seq
    ...............................augcgggaucuuuaguuguggcC...........................................................   3       1    seq
    ................................ugcAggaucuuuaguugu...............................................................   2       1    seq
    ................................ugcgggaucuuuaguugA...............................................................   6       1    seq
    ................................ugcgggaucuuuaguugu...............................................................   56      0    seq
    ................................ugUgggaucuuuaguugug..............................................................   1       1    seq
    ................................ugcgggaucuuuaguugug..............................................................   85      0    seq
    ................................ugcgggaucuuuaguuguA..............................................................   26      1    seq
    ................................ugcAggaucuuuaguugug..............................................................   1       1    seq
    ................................ugcCggaucuuuaguugug..............................................................   1       1    seq
    ................................ugUgggaucuuuaguugugg.............................................................   2       1    seq
    ................................ugcUggaucuuuaguugugg.............................................................   1       1    seq
    ................................ugcCggaucuuuaguugugg.............................................................   8       1    seq
    ................................ugcgggaucuuuaguugugU.............................................................   34      1    seq
    ................................ugcgggGucuuuaguugugg.............................................................   1       1    seq
    ................................ugcgggaucuuuagCugugg.............................................................   2       1    seq
    ................................uAcgggaucuuuaguugugg.............................................................   2       1    seq
    ................................ugUgggaucuuuaguuguggc............................................................   2       1    seq
    ................................ugcgggaucuuuaguugugUc............................................................   1       1    seq
    ................................ugcgggaucuuuaguugCggc............................................................   2       1    seq
    ................................ugcAggaucuuuaguuguggc............................................................   6       1    seq
    ................................ugcgggGucuuuaguuguggc............................................................   8       1    seq
    ................................ugcCggaucuuuaguuguggc............................................................   7       1    seq
    ................................ugcgggaucuuuaguuguggc............................................................   906     0    seq
    ................................ugcggAaucuuuaguuguggc............................................................   1       1    seq
```

Mature **Star**

```
auaacccauaaaaagcagccgacguugcggcaugcgggaucuuuaguuguggcgugugcauucuuaguugcagcaacuaaagaucccucaggcugcaaugaagaucagagauc
................................ugcgggaucuuuaguuguggU............................................................ 468   1  seq
................................ugcUggaucuuuaguuguggc............................................................ 1     1  seq
................................uAcgggaucuuuaguuguggc............................................................ 2     1  seq
................................ugcgggGucuuuaguuguggcg........................................................... 12    1  seq
................................ugcgggaucuuuagCuguggcg........................................................... 6     1  seq
................................ugcUggaucuuuaguuguggcg........................................................... 1     1  seq
................................ugcAggaucuuuaguuguggcg........................................................... 21    1  seq
................................ugcgggaucuuuaguuguggcg........................................................... 2940  0  seq
................................uAcgggaucuuuaguuguggcg........................................................... 16    1  seq
................................ugcgggaucuuuaguuguggcgu.......................................................... 2440  0  seq
................................ugcgggGucuuuaguuguggcgu.......................................................... 11    1  seq
................................ugcgggaucuuuagCuguggcgu.......................................................... 6     1  seq
................................uAcgggaucuuuaguuguggcgu.......................................................... 15    1  seq
................................ugcgggaucuuuaguuguggcCug......................................................... 1     1  seq
................................ugcgggaucuuuaguuguggcgugu........................................................ 3     0  seq
.......................................................................agcaacuaaagaucccucagg..................... 3     0  seq
.......................................................................agcaacuaaagaucccucaggc.................... 7     0  seq
```
